# Myxoid Malignant Fibrous Histiocytoma with Multiple Primary Sites

**DOI:** 10.1080/13577140220127567

**Published:** 2002-03

**Authors:** Jeffrey H. Muler, Augusto F. Paulino, Diane Roulston, Laurence H. Baker

**Affiliations:** 1 Department of Hematology/Oncology University of Michigan Ann Arbor MI USA; 2 Department of Pathology University of Michigan Ann Arbor MI USA; 3 University of Michigan Cancer Center 1500 east Medical Center Drive 7216 CCCGC Ann Arbor MI 48109-0948 USA

## Abstract

Malignant fibrous histiocytoma (MFH) is one of the most common types of soft tissue sarcomas in adults. The most common location of MFH are the extremities and the trunk, with the most common site for distant metastases being the lung. We describe a case with multiple synchronous sites of myxoid MFH but no lung metastases and presence of abnormalities of 19p13.


*Sarcoma* (2002) **6**, 51–55

Taylor & Francis healthsciences

**CASE REPORT**

### Myxoid malignant fibrous histiocytoma with multiple primary sites

JEFFREY H. MULER[1], AUGUSTO F. PAULINO[2], DIANE ROULSTON[2] & LAURENCE H. BAKER[3]

*Departments of [1]Hematology/Oncology and [2]Pathology, University of Michigan, and [3]University of Michigan Cancer Center, Ann Arbor, MI, USA*

**Abstract**

Malignant fibrous histiocytoma (MFH) is one of the most common types of soft tissue sarcomas in adults. The most common location of MFH are the extremities and the trunk, with the most common site for distant metastases being the lung. We describe a case with multiple synchronous sites of myxoid MFH but no lung metastases and presence of abnormalities of 19p13.

### Introduction

Malignant fibrous histiocytoma (MFH) is one of the most common types of soft tissue sarcomas in adults,[1,2] with the majority of tumors arising in the trunk or the extremities. About 30–40% of patients with MFH develop distant metastases, with the most common site being the lung.[3–7] Metastatic disease in the absence of lung metastases is highly unusual. This report describes a case in which a patient had multiple synchronous myxoid MFH tumors without evidence of lung metastases. We believe that the patient had multiple primary sites of myxoid MFH, thus raising the possibility of a genetic abnormality that could predispose such a patient to develop multiple sites of the same tumor. The description of the tumor karyotype in the case of our patient is provided.

### Case report

The patient was a 67-year-old man who originally noticed a lump in his right calf area 3 years previously. Magnetic resonance imaging at that time confirmed a tumor in the right calf, and a core needle biopsy revealed high-grade myxoid malignant fibrous histiocytoma (MFH) (Fig. 1). The patient was treated with neoadjuvant chemotherapy (mesna, doxorubicin, ifosfamide and dacarbazine) and underwent tumor resection followed by adjuvant radiation. He remained well until 2 years later when he noticed two 10-cm lumps in the upper right thigh and pain in the epigastric area. A computerized tomography scan of the abdomen performed at that time revealed a gastric mass, and the patient underwent excision of the two thigh tumors followed by partial gastrectomy. The pathology from all three sites was reviewed and revealed high-grade myxoid MFH in each of them (Figs. 2 and 3). The surgical margins of the two thigh lesions resected were negative. The CD117 (c-kit) stains performed on the partial gastrectomy specimen were negative. The patient remained well for 3 months, when he presented to our institution with a rapidly enlarging mass on the right forearm. Staging CT scans preoperatively revealed no evidence of pulmonary or abdominal metastasis and patient underwent wide excision of the right forearm lesion, which again turned out to be high-grade myxoid MFH.

Cytogenetic analysis was performed on the fresh samples from the right forearm excision. An abnormal clone with complex cytogenetic abnormalities was observed, that had the following hypodiploid karyotype: 39, der(X;6)idic(6)(q12)t(6;8)(p23;q13) t(X;6)(p22.1;p23),-Y,-1,der(1)t(1;15)(p31;q13),der (3;9)t(3;9)(p26;q34)add(9)(p24), i(4)(p10),der(6) t(2;6)(q31;q27)ins(6;?)(q27;?),add(7)(q32),der(7) t(7;?11)(q36;q21)ins(7;?)(q36;?),-8, -8,-10,del(11) (p11.1p15.5),-13,-13,+14,der(14) t(3;14)(p21; q32),-15,der(15)t(15;?22)(p11.2;q11.2),-17,-17,-18,ins(18;?)(p11.2;?),add(19)(p13.1),del(19) (p13.2p13.3),+22,del(22)(q13.1q13.3),der(22)del

Correspondence to: Laurence Baker, D.O., University of Michigan Cancer Center, 1500 east Medical Center Drive, 7216 CCCGC, Ann Arbor, MI 48109–0948, USA. Tel.: +1–734–936–3983; Fax: +1–313–936–7376; E-mail: bakerl@umich.edu

1357–714X print/1369–1643 online/02/010051–05 © 2002 Taylor & Francis Ltd
DOI: 10.1080/1357714022012756 7

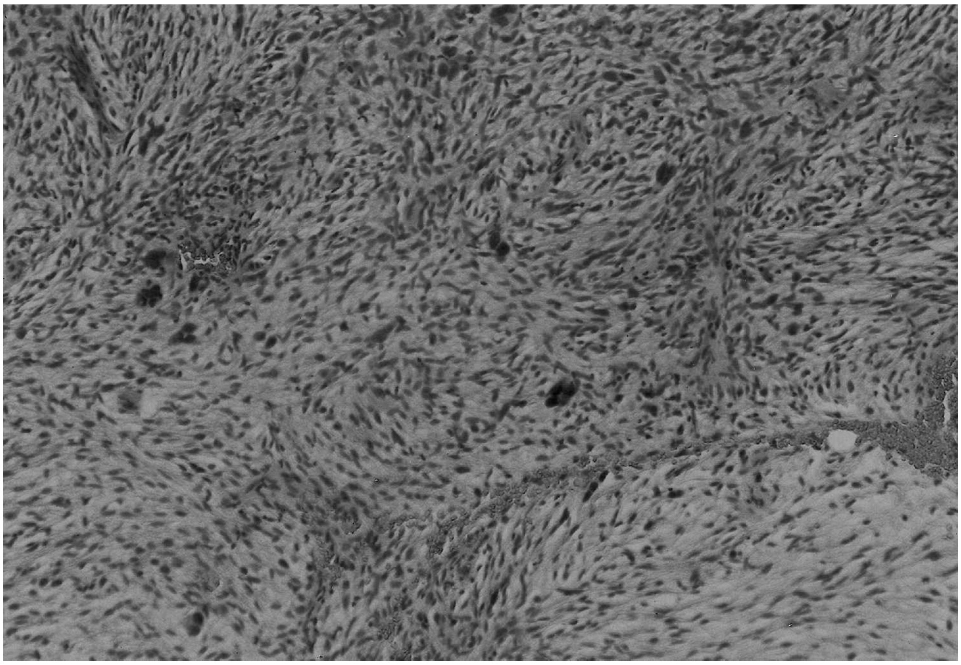

**Fig. 1.** *H&E stain right calf biopsy, ×200.*

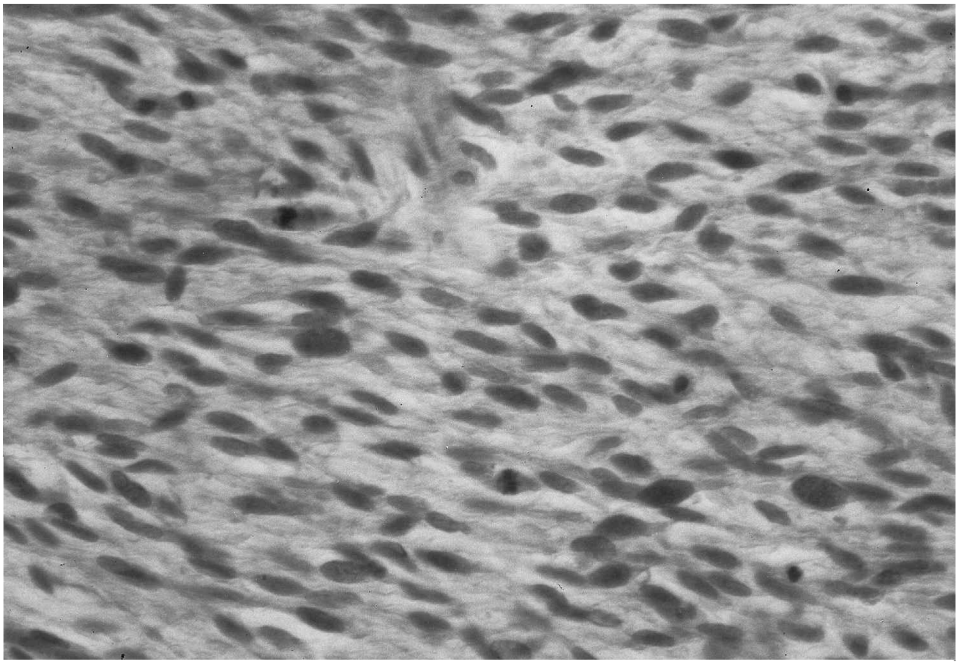

**Fig. 2.** *H&E stain right thigh resection, ×400.*

(22)(p11.1p13)del(22)(q12q13),+ mar 1, + mar 2, + mar 3.

### Discussion

Malignant fibrous histiocytoma (MFH) is considered to be one of the most common types of soft tissue sarcomas in adults, accounting for 36–40% of all soft tissue sarcomas analyzed in two large series.[1,2] The majority of MFH arise in the proximal extremities or the trunk.[2–4] The incidence of MFH increases with age, with the majority of patients being over 50 years of age.[4] The local recurrence rate for MFH is 28–51% depending on whether adjuvant radiation was used.[2–6] The rate of distant metastasis varies from 30 to 46%.[2,3,5,7] The most common site of distant metastasis was by far the lung (63–91%), followed by lymph nodes (10%) and bone (3–8%), taken as a proportion of all metastatic sites.[3,4,7] Table 1 illustrates the overall survival (OS) and disease-free survival (DFS) for MFH from various reported series:

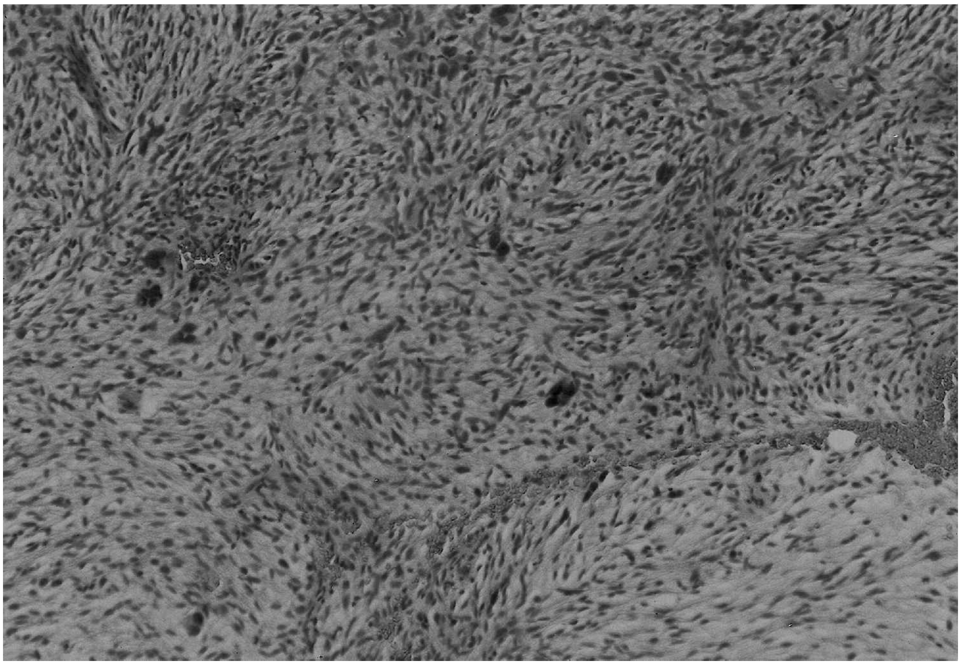

**Fig. 3.** *H&E stain mass from partial gastrectomy, ×400.*

**Table 1.** *Overall and disease-free survival in MFH*

| Ref. | OS at 5 years (%) | DFS at 5 years (%) | Median follow-up (months) |
|---|---|---|---|
| 4 | 67.2 | 50.6 | 73 |
| 5 | 58 | 36 | 42 |
| 7 | 36 | NA | 72 |
| 8 | 70 | NA | 36 |

*Abbreviations*: OS, overall survival; DFS, disease-free survival.

**Table 2.** *Significant prognostic factors in MFH*

| Factor | Outcome influenced | Ref. |
|---|---|---|
| Tumor size | MFS, OS | 2,3,4,5,7,8 |
| Subtype: myxoid versus non-myxoid | MFS, DSS, OS | 2,3,8 |
| Tumor necrosis | MFS, OS | 2,8 |
| UICC Stage III+IVA | DSS | 3 |
| Deep tumor location | DSS | 3,6 |
| Age > 50 | DSS | 3 |
| Grade | MFS, OS | 3,4,5 |
| Negative margins after primary resection | LRFS, DFS, OS | 3,5,7 |

*Abbreviations*: MFS, metastasis free survival; DSS, disease-specific survival; OS, overall survival; DFS, disease-free survival; LRFS, local recurrence-free survival.

A number of prognostic factors have been examined in terms of their predictive significance for overall survival and recurrence in MFH by various investigators, in an attempt to better define different prognostic variables in this diverse group of soft tissue sarcomas (Table 2).

There are four major variants of MFH recognized today: storiform-pleomorphic (the most common), myxoid, giant cell and inflammatory.[9] The myxoid type of MFH (also known as myxofibrosarcoma) accounts for about a third of all MFH cases.[2,9] This subtype of MFH was first described by several authors in the late 1970s, and was based on its distinct gelatinous appearance grossly, with a variable amount of myxoid stroma adjacent to the cellular areas microscopically.[10–12]

The median age at diagnosis of myxoid MFH is 65 years[13] with peak incidence at 60–69 years.[11] The most common primary site was the extremities (77%), the trunk (15%) and the retroperitoneum (8%).[13] In 70% of cases, the primary tumor was subcutaneous at presentation.[13] It has been argued that the myxoid type represents a distinct subgroup of MFH based on its better prognosis compared to the other MFH

variants.[12] The reported rates of recurrence vary from 52 to 66%.[11,13] Metastasis occurred in 23–35% of cases, with the most common site of metastasis being the lungs, followed by the lymph nodes, skeleton and liver.[11,13] Mentzel *et al.* have described a low-grade variant of myxofibrosarcoma, with an even lower incidence of distant metastasis.[14] Within this group, the authors described five cases in which the low-grade myxofibrosarcoma tended to become progressively higher grade with each recurrence.[14]

Factors that are believed to favorably influence recurrence rates and metastasis in myxoid MFH include: small size, superficial location, increased proportion of the myxoid component and low grade.[11,14] In the original Scandinavian series on myxoid MFH, the 5- and 10-year survival rates were 65 and 52%, respectively.[13] Histological grade, type of surgical therapy, tumor size, age, and interval to first recurrence were important predictors for survival and recurrence.[13]

The case reported here has an unusual feature, in that the patient may have had what we believe to be multiple new primary sites of MFH in locations such as soft tissues of the extremities, without evidence of metastases to the lung parenchyma.

There are two possible explanations for such an unusual clinical course. It is conceivable that the reason our patient did not have lung metastases is because the multiple recurrences represent separate myxoid MFH primary sites. The superficial location of the patient's lesions would place him at a lower risk for distant metastases, as superficial location of the tumor had a higher local recurrence rate but a decreased incidence of distant metastases compared to the deep lesions.[3,6,11] Myxoid MFH primary tumors have been known to present as superficial lesions in up to 70% of cases.[13,14] An alternative explanation is that the patient developed multiple subcutaneous metastases over the clinical course of the disease. Coincidental second primary tumors previously described with myxoid MFH include: palmar fibromatosis, lipomas, basal cell carcinomas of the skin, carcinomas of the large bowel and stomach, and carcinomas of the ovary and uterus.[13] However, only one case in the early original series on myxoid MFH described a patient with multiple synchronous tumors on the arm, buttock and shoulder.[11]

The absence of ring chromosomes and the presence of 19p+ marker in our case would be consistent with an increased relapse rate, both locally and distally, and would support the distant metastases point of view.[20,21]

None of the above arguments disprove that the multiple lesions could have represented distant metastases. After all, our myxoid MFH lesions were high grade with a small myxoid component, a finding associated with a 24–31% rate of distant metastasis.[11] However, such a pattern without pulmonary involvement is highly unusual. An early series on myxoid MFH described two patients with a similar clinical course of multiple subqutaneous recurrences and involvement of the GI tract.[10] Both patients developed pulmonary metastases, while the patient in our case did not.

Despite its being one of the most common types of soft tissue sarcomas in many published clinical series, the validity of classifying certain types of MFH as separate histopathological entities has been questioned.[15,16] In a recent study by Fletcher *et al.*,[17] of 61 cases initially labeled as storiform–pleomorphic MFH, all but one case was reclassified using predefined histopathological criteria proposed by the authors. Myxoid MFH (myxofibrosarcoma) on the other hand, remained the most common subtype in that series. Thus, it appears that myxoid MFH is one subtype of MFH that represents a distinct reproducible entity.[18]

Advances in various techniques of chromosomal analysis have prompted many investigators to try and define MFH on a more basic molecular level. The earlier studies revealed a clonal chromosomal abnormality in 17 out of 25 cases of MFH, with further observation that the presence of an abnormal $19p^{+}$ marker chromosome was associated with increased frequency of local recurrence.[19–21] Breakpoints in 1q11, 1p36, 3p12, 11p11 and 19p13 were frequently observed.[19] Another group reported two cases with sole abnormalities of t(5;7)(q31;q22) and der(13;14)(q10;q10), raising the possibility that they represent primary cytogenetic abnormalities.[22] Using the technique of comparative genomic hybridization, investigators in one study were able to demonstrate that gain of 7q32 was associated with decreased metastasis-free survival and overall survival, whereas gain of 1p31 was associated with a trend towards decreased overall survival.[23]

The cytogenetic findings in our case revealed abnormalities of 19p13 for both of the homologues that were present, an abnormality reported to have adverse prognostic significance with increased rates of recurrence and metastasis.[19–21] A trend towards fewer relapses in tumors with ring chromosomes was noted in one of the series.[21] Of note, no ring chromosomes were found in our case. Previously reported abnormalities also found in our case include: presence of hypodiploid clone, abnormality of 9p and 11p11, and presence of dicentric chromosomes der(X;6), der(3;9) are dicentric.

Several candidate genes implicated in the pathogenesis of MFH have been recently identified. Frequent amplification in the region of 8p23.1 in eight out of 14 MFHs has led one group of investigators to identify a novel gene *MASL1* located in this narrow region.[24] The product of this gene is believed to be an ATP/GTP-binding protein involved in the regulation of the cell cycle. Frequent loss of region 13q12–q14 and 13q21, reported in 78% of MFHs analyzed in one series, led the authors to believe that mutation or inactivation of the *RB1* tumor suppressor gene could be an initial event in the pathogenesis of MFH.[25,26] Mutations of *TP53* tumor suppressor gene were also demonstrated in series on MFH.[27,28] Finally, presence of a shared deletion of 9p21 between a rare inherited form of MFH and sporadic MFH indicated that the tumor suppressor genes present in that location *CDKN2A/B* could contribute to tumorigenesis in MFH.[29] This hypothesis is supported by one recent study, which identified frequent loss of 9p21 leading to the deletion of the *CDKN2A* gene in up to 55% of MFH cases analyzed.[28]

Of note, however, no single cytogenetic or molecular rearrangement exists that has been shown to be pathognomonic of MFH. It has been argued that the broad range of complex rearrangements frequently reported with MFH stems from the possibility that the cases represent groups of heterogeneous sarcomas.[22]

In conclusion, we report a case of myxoid MFH with possibly multiple primary sites, absence of pulmonary metastases and presence of an abnormality of 19p13. This, in turn, raises the possibility of a yet undetermined germ line mutation specific for MFH in such a patient, that could account for the development of multiple primary sites of the same tumor. It also demonstrates that within the spectrum of MFH, subgroups of patients may have a distinct clinical course based on the different molecular pathogenesis of certain types of MFH. Further studies aimed at determining the exact cellular aberrations associated with this neoplasm and their clinical significance are warranted.

### References

1. Coindre JM, Terrier P, Binh Bui N, *et al.* Prognostic factors in adult patients with locally controlled soft tissue sarcoma: a study of 546 patients from the French Federation of Cancer Centers Sarcoma Group. *J Clin Oncol* 1996; 14: 869–77.
2. Gustafson P. Soft tissue sarcoma: epidemiology and prognosis in 508 patients. *Acta Orthopaedica Scand* 1994; 65 (Suppl 259): 1–29.
3. Le Doussal V, Coindre JM, Leroux A, *et al.* Prognostic factors for patients with localized primary malignant fibrous histiocytoma: a multicenter study of 216 patients with multivariate analysis. *Cancer* 1996; 77: 1823–30.
4. Pezzi CM, Rawlings MS, Esgro JJ, *et al.* Prognostic factors in 227 patients with malignant fibrous histiocytoma. *Cancer* 1992; 69: 2098–103.
5. Gibbs JF, Huang PP, Lee JR, *et al.* Malignant fibrous histiocytoma: an institutional review. *Cancer Invest* 2001; 19(1): 23–7.
6. Kearney MM, Soule EH, Ivins JC. Malignant fibrous histiocytoma: a retrospective study of 167 cases. *Cancer* 1980; 45: 167–78.
7. Bertoni F, Capanna R, Biagini R, *et al.* Malignant fibrous histiocytoma of soft tissue. *Cancer* 1985; 56: 356–67.
8. Rooser B, Willen H, Gustafson P, *et al.* Malignant fibrous histiocytoma of soft tissue. *Cancer* 1991; 67: 499–505.
9. Enzinger FM, Weiss SW. *Soft Tissue Tumors*, 3rd ed. St Louis, MO: Mosby, 1995; 351–80.
10. Angervall L, Kindblom LG, Merck C. Myxofibrosarcoma. *Acta Pathol Microbiol Scand Sect A* 1977; 85: 127–40.
11. Weiss SW, Enzinger FM. Myxoid variant of malignant fibrous histiocytoma. *Cancer* 1977; 39: 1672–85.
12. Weiss SW. Malignant fibrous histiocytoma. A reaffirmation. *Am J Surg Pathol* 1982; 6: 773–84.
13. Merck C, Kindblom LG, Oden A. Myxofibrosarcoma: a malignant soft tissue tumor of fibroblastic-histiocytic origin. *Acta Pathol Microbiol Scand Sect A* 1983; 91 (Suppl 282): 1–40.
14. Mentzel T, Calonje E, Wadden C, *et al.* Myxofibrosarcoma: clinicopathologic analysis of 75 cases with emphasis on the low-grade variant. *Am J Surg Pathol* 1996; 20: 391–405.
15. Fletcher CDM. Malignant fibrous histiocytoma? *Histopathology* 1987; 11: 433–7.
16. Fletcher CDM. Pleomorphic malignant fibrous histiocytoma: Fact or fiction? A critical reappraisal based on 159 tumors diagnosed as pleomorphic sarcoma. *Am J Surg Pathol* 1992; 16: 213–28.
17. Fletcher CDM, Gustafson P, Rydholm A, *et al.* Clinicopathologic re-evaluation of 100 malignant fibrous histiocytomas: prognostic relevance of subclassification. *J Clin Oncol* 2001; 19: 3045–50.
18. Hollowood K, Fletcher CDM. Malignant fibrous histiocytoma: morphologic pattern or pathologic entity? *Semin Diagn Pathol* 1995; 12: 210–20.
19. Mandahl N, Heim S, Willen H, *et al.* Characteristic karyotypic anomalies identify subtypes of malignant fibrous histiocytoma. *Genes Chromosomes Cancer* 1989; 1: 9–14.
20. Rydholm A, Mandahl N, Heim S, *et al.* Malignant fibrous histiocytoma with 19p+ marker chromosome have increased relapse rate. *Genes Chromosomes Cancer* 1990; 2: 296–9.
21. Choong PFM, Mandahl N, Mertens F, *et al.* 19p+ Marker chromosome correlates with relapse in malignant fibrous histiocytoma. *Genes Chromosomes Cancer* 1996; 16: 88–93.
22. Walter TA, Weh HJ, Schlag PM, *et al.* Cytogenetic studies in malignant fibrous histiocytoma. *Cancer Genet Cytogenet* 1997; 94: 131–4.
23. Larramendy ML, Tarkkanen M, Blomqvist C, *et al.* Comparative genomic hybridization of malignant fibrous histiocytoma reveals a novel prognostic marker. *Am J Pathol* 1997; 151: 1153–61.
24. Sakabe T, Shinomiya T, Mori T, *et al.* Identification of a novel gene, MASL1, within an amplicon at 8p23.1 detected in malignant fibrous histiocytomas by comparative genomic hybridization. *Cancer Res* 1999; 59: 511–5.
25. Chibon F, Mairal A, Freneaux P, *et al.* The RB1 gene is the target of chromosome 13 deletions in malignant fibrous histiocytoma. *Cancer Res* 2000; 60: 6339–45.
26. Mairal A, Terrier P, Chibon F, *et al.* Loss of chromosome 13 is the most frequent genomic imbalance in malignant fibrous histiocytoma. *Cancer Genet Cytogenet* 1999; 111: 134–8.
27. Taubert H, Wurl P, Meye A, *et al.* Molecular and immunohistochemical p53 status in liposarcoma and malignant fibrous histiocytoma. *Cancer* 1995; 76: 1187–96.
28. Simons A, Schepens M, Jeuken J, *et al.* Frequent loss of 9p21 (p16INK4A) and other genomic imbalances in human malignant fibrous histiocytoma. *Cancer Genet Cytogenet* 2000; 118: 89–98.
29. Martignetti JA, Gelb BD, Pierce H, *et al.* Malignant fibrous histiocytoma: inherited and sporadic forms have loss of heterozygosity at chromosome bands 9p21–22 — evidence for a common genetic defect. *Genes Chromosomes Cancer* 2000; 27: 191–5.
